# Age related differences in acute coronary syndrome presentation and in hospital outcomes: a cross-sectional comparative study

**DOI:** 10.11604/pamj.2016.24.337.8711

**Published:** 2016-08-31

**Authors:** Hyder Osman Mirghani

**Affiliations:** 1 Faculty of Medicine, University of Tabuk, Tabuk, Kingdom of Saudi Arabia

**Keywords:** Age, differences, acute, coronary, presentation, in Hospital, outcomes, Sudan

## To the editors of the Pan African Medical Journal

Coronary artery disease is prevalent worldwide and causing much morbidity and mortality, it causes 12.7% of death across the earth; the burden is involving all countries and communities [1]. Elderly patients with the acute coronary syndrome are at particular risk of death and morbidity, 83% of acute coronary syndrome mortality occur in patients ≤65 years [2]. We aimed to assess age-related differences in acute coronary syndrome presentation, management, and in-hospital complications. A comparative cross-sectional study conducted at a coronary care unit in Omdurman Teaching Hospital, Sudan. The study included 202 patients older than 18 years with the diagnosis of the acute coronary syndrome from July 2014-August 2015. Patients were invited to sign a written informed consent, and then a case report form including demographic data, hypertension, diabetes, smoking, prior myocardial infarction, reperfusion therapy, and in-hospital complications was filled. The ethical committee of Omdurman Teaching Hospital approved the research, and the Statistical Package for Social Sciences was used with P-value of < 0.05 considered significant. Comparison between patients below 50 years and those aged 50 or older was undertaken using the chi-square test. Older patient with the acute coronary syndrome were more likely to be hypertensive (57.6%vs.41.4%), had heart failure ( 56.2%vs,37.2%), presented to hospital>12 hours after onset of pain, less likely to receive thrombolytic therapy (9.2%vs. 23.5%), and had more in-hospital complications (64.2%vs.45.1%) P-value < 0.05. No statistical differences were evident between young and old patients regarding coronary risk factors, coronary angiography, and hypotension P-value > 0.05. Elderly acute coronary syndrome patients were more likely to have hypertension, heart failure, and in-hospital complications, they presented later to the hospital, and less likely to benefit from thrombolytic therapy.

Elderly patients are more likely to have complications both from acute coronary syndrome and reperfusion therapy due to more vascular risk factors, frailty, and age-related physiological changes. Furthermore, the expected mortality rate is higher. In spite of the American College of Cardiology/American Heart Association (ACC/AHA) guidelines statement that age should not influence patients cares elderly acute coronary syndrome patients benefit less from evidence-based therapy including interventional procedures [3, 4]. There is an increasing awareness of the clinical presentation, ECG, and outcomes of acute coronary syndrome among elderly people due to expanding aging populations [5]. Sudan is a vast country with ethnic and socio-demographic diversity, the mean life expectancy is 62.9 years [6], so the clinical presentation and outcome of acute coronary syndrome may be different from those in developed countries. We investigated 200 with the diagnosis of acute coronary syndrome at the coronary care unit in Omdurman Teaching Hospital, Sudan from July 2014 to August 2015. Patients signed a written informed consent and a case report form was filled including, Age, sex, ST-Segment Elevation Myocardial Infarction (STEMI), and None ST-Segment Acute Coronary Syndrome (NSTEMI and Unstable Angina collectively), past history of myocardial infarction, diabetes, hypertension, smoking, presentation of acute coronary syndrome, the time before presentation at hospital, the pulse rate, hypotension, heart failure, echocardiography report, thrombolytic therapy and coronary angiography. The diagnosis of the acute coronary syndrome was based on typical chest pain, ECG changes, and elevated cardiac biomarkers. A blood sample at admission was taken for urea, creatinine, cholesterol, and triglyceride estimation. In-hospital complications including arrhythmias (reperfusion arrhythmias were not counted), cardiogenic shock, and death were recorded. Study population was divided into two categories as 18 to 50 years of age and above 50 years of age in order to compare all the variables. Out of 202 acute coronary syndrome patients, male dominance was apparent. STEMI was more common in males patient in the young age group (52.9%vs.47.1%) while it was commoner in females among the older age group (45.6%vs.54.4%) P value=0.419. No statistically significant difference regarding coronary syndrome risk factors apart from hypertension which was evident in 41.4% of the young age group and 57.6% of the older age group P-value=0.05.

**Figure 1:**
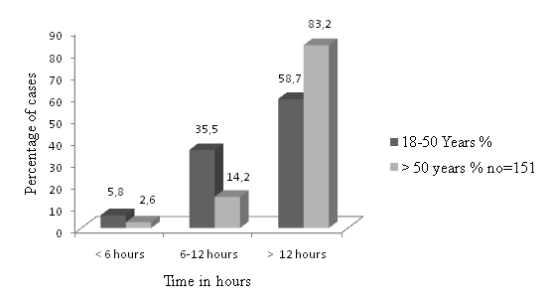
Duration of chest pain

The hospital time course of the acute coronary syndrome patients showed: pulse abnormalities were more evident in elder age group with no statistically significant difference P > 0.05. Hypotension and heart failure were more common in the older age group (19.6%vs 27.1%) P-value=0.723, and (1.6% vs. 50.3%) respectively P-value=0.023. Older patients were less likely to receive thrombolytic therapy (9.2%vs.23.5%) P-value=0.009, only minority of this sample had undergone coronary angiography (7.8% of young vs.12.5% of old patients) P-value=0.603. Low ejection fraction was found in 54.8% of young patient vs. 67.5% of the old acute coronary syndrome patients P-value=0.692 (Table 1). The majority of the patients with chest pain present to the hospital more than 12 hours illustrated in the Figure 1. In the present study ST-Segment Elevation Myocardial Infarction was more prevalent among the younger age group in agreement with Stem et al. [5]. Hypertension was commoner among older patients and in agreement with Ahmed et al. [3]. No significant statistical difference was found regarding diabetes mellitus, and prior myocardial infarction one plausible explanation is the closer age ranges in the two subgroups as only 12.6% of our samples were above 70 years old. It is interesting to show that: the majority (83.2%) of older group presented at hospital later than 12 hours, difficulties with transportation, and traffic congestion may explain the delay, lack of awareness among the public about the seriousness of chest pain may be a contributing factor, also in some regions of our country people prefer to manage the very ill old patients at home instead of transferring them to hospitals due to the belief that the result is the same. In the current study patient had higher in-hospital complications than their other counterparts [7]. Furthermore, the older subgroup was less likely to receive thrombolytic therapy than the younger subset. This paradox may be due to physician fear regarding outcomes especially in elderly female [8, 9]. Regarding coronary angiography it is less than the rate observed in South Africa and Germany [7, 10]. The rate of PCI is apparently depending on the patient presentation at hospital, in the current study the majority of patients presented to the hospital more than the suitable time for PCI. The Statistical Package for the Social Sciences software SPSS version 20 was used for data analysis. The Chi-square test was used to compare categorical data with a P. Value equal or less than 0.05 accepted for statistical significance.

**Table 1:** Comparison between the participants aged 18-50 years, and above 50 years

Character	18-50 Years % n=51	> 50 years % n=151	P-value
Prior myocardial infarction	13.7	21.8	0.208
Diabetes mellitus	29.4	30.4	0.888
Hypertension	41.4	57.6	0.050
Smoking	21.5	17.8	0.086
Ischemic chest pain	92	97.3	0.044
Tachycardia	39.2	49	0.390
Irregular pulse	17.6	27.1	0.390
Bradycardia	3.9	5.3	0.390
Hypotension	19.6	27.1	0.723
Heart failure	15.6	50.3	0.023
Thrombolysis	23.5	9.2	0.009
Coronary angiography	7.8	12.5	0.603
Low ejection fraction	54.8	67.5	0.692
Intraventricular thrombus	3.9	5.9	0.734
Hypertriglyceridemia	13.7	17.2	0.665
Hypercholesterolemia	13.7	19.2	0.526
Renal impairment	21.5	19.8	0.841
**In-hospital complications:**	45.1	64.2	0.016
Arrhythmias	21.5	33.1	
Heart failure	15.6	50.3	
Cardiogenic shock	9.8	11.2	
Death	1.9	5.2	

1 Some patients had more than one complication

The current data highlighted that Sudanese acute coronary syndrome patients did not present to the Hospital at the early right time for intervention, and elderly people benefited less from reperfusion therapy. Our data can be used to develop health promotion in Sudan. Physicians should strictly adhere to guidelines, especially for older coronary syndrome patients, larger multicenter studies are needed to assess obstacles to acute coronary care in Sudan.
